# Dietary Intake and Anthropometric Measurement at Age 36 Months Among Aboriginal and/or Torres Strait Islander Children in Australia: A Secondary Analysis of the Baby Teeth Talk Randomized Clinical Trial

**DOI:** 10.1001/jamanetworkopen.2021.14348

**Published:** 2021-07-08

**Authors:** Lisa Gaye Smithers, Joanne Hedges, Pedro Henrique Ribeiro Santiago, Lisa M. Jamieson

**Affiliations:** 1 University of Adelaide School of Public Health, Adelaide, South Australia, Australia; 2 University of Wollongong School of Health and Society, Wollongong, New South Wales, Australia; 3 Australian Research Centre for Population Oral Health, Adelaide Dental School, University of Adelaide, Adelaide, South Australia, Australia

## Abstract

**Importance:**

Interventions to reduce early childhood caries should be examined for their effects on anthropometry given their design to improve children’s diets.

**Objective:**

To compare the outcomes of dietary intake, anthropometric measurements, and blood pressure measurements between children at age 36 months in the immediate intervention group vs those in the delayed intervention group.

**Design, Setting, and Participants:**

This secondary analysis was a follow-up to the 2-group Baby Teeth Talk randomized clinical trial conducted across the state of South Australia, Australia. Participants were Aboriginal and/or Torres Strait Islander children and their caregivers who were randomized to the immediate intervention group or delayed intervention group. The intervention was provided from February 1, 2011, to May 31, 2012. The prespecified follow-up when the participating children were aged 36 months was conducted from November 1, 2014, to February 28, 2016, in participant homes or public locations. Data were analyzed from October 5, 2018, to April 29, 2019.

**Interventions:**

The immediate intervention group received the intervention during pregnancy and at 6, 12, and 18 months of age. The delayed intervention group received the intervention at 24, 30, and 36 months of age. Both groups received an intervention consisting of free dental care for mothers, fluoride varnish on children’s teeth, anticipatory guidance on oral health and dietary advice, and motivational interviewing.

**Main Outcomes and Measures:**

Dietary intake was measured with a caregiver-completed, 17-item food frequency questionnaire. Frequency of consumption of discretionary foods and beverages were the main dietary outcomes. Children’s weight, height, and mid–upper arm circumference were measured and converted to age- and sex-specific *z* scores. Body mass index *z* score was the main anthropometric outcome.

**Results:**

A total of 330 children were followed up to age 36 months among the 448 mothers and 454 children who were randomized to the 2 groups. At baseline, the women had a mean (SD) age of 24.9 (5.9) years, and the children had a mean (SD) weight of 3.3 (0.6) kilograms at birth, and 205 were boys (46%); sex was not recorded for 63 children (14%). Diet outcomes were similar between the groups. For example, the mean (SD) intake of discretionary beverages by the immediate intervention group was similar to that by the delayed intervention group (507 [536] mL/d vs 520 [546] mL/d; adjusted mean difference [MD], −16 [95% CI, −133 to 102] mL/d; *P* = .79). Height was similar between the 2 groups, but the mean (SD) *z* scores of weight (0.7 [1.0] vs 0.4 [1.0]; adjusted MD, 0.3 [95% CI, 0.1-0.5]; *P* = .02), arm circumference (1.6 [1.0] vs 1.3 [0.9]; adjusted MD, 0.2 [95% CI, 0.1-0.5]; *P* = .03), and body mass index (1.1 [1.1] vs 0.9 [0.9]; adjusted MD, 0.2 [95% CI, 0.0-0.4]; *P* = .04) were higher in the immediate intervention group than the delayed intervention group.

**Conclusions and Relevance:**

This study found no differences in dietary intakes between children who received an intervention to reduce dental caries early and those who received it later. At age 36 months, children in the immediate intervention group had greater *z* scores for weight, arm circumference, and body mass index than their counterparts in the delayed intervention group, suggesting a potential implication of oral health interventions for anthropometric outcomes.

**Trial Registration:**

ANZCTR Identifier: ACTRN12611000111976

## Key Points

**Question:**

What is the effect of providing an intervention to prevent childhood dental caries earlier (during pregnancy and at ages 6, 12, and 18 months) rather than later (aged 24, 30, and 36 months) on intake of unhealthy foods and body mass index among Aboriginal and/or Torres Strait Islander children at age 36 months?

**Findings:**

In this secondary analysis of 448 mothers and 454 children enrolled in a randomized clinical trial, no differences in discretionary food intake were found between 2 groups of children, but those who received the intervention earlier had greater *z* scores for weight, arm circumference, and body mass index than children who received the intervention later.

**Meaning:**

Findings from this analysis suggest that an oral health intervention could have unintended consequences for children, such as worse anthropometric outcomes.

## Teaser

This secondary analysis reports the follow-up results of a dental care service provided to Indigenous Australian children and their caregivers who participated in the Baby Teeth Talk randomized clinical trial.

## Introduction

The diets of young children have been linked to health outcomes, such as obesity and high blood pressure.^1,2^ Often, poor diets begin in infancy and continue over time. In Australia, 41% of the total dietary energy consumed by 1- to 2-year-old children is from unhealthy foods,^3^ with similarly high intakes reported among toddlers in the United States.^4,5^ Consequently, there has been much interest in interventions that might set children on healthier dietary trajectories. However, to date, most randomized clinical trials (RCTs) of interventions to improve the diets of young children have reported small or null effects on diet or anthropometry.^6,7,8^

It is not clear at which age in childhood to introduce interventions for dietary and medical conditions of future concern to public health, such as high anthropometric measurements and dental caries. In an RCT (Baby Teeth Talk trial) that was conducted among families with Australian Aboriginal and/or Torres Strait Islander children, the multifaceted, culturally safe intervention involved oral health care for mothers, motivational interviewing, anticipatory guidance, and fluoride varnish on children’s teeth.^7,9^ The trial focused on Aboriginal and/or Torres Strait Islander children because they experience a range of health disparities across the lifespan from higher mortality at birth to lower life expectancy^10^ compared with non-Aboriginal and/or Torres Strait Islander children and because progress has been slow on improving the health of Aboriginal and/or Torres Strait Islander populations.^11^

In the trial, the intervention group received the intervention at 4 time points from pregnancy to 18 months of age, whereas the delayed intervention group received the intervention at 3 time points at child age 24, 30, and 36 months. The primary outcome at age 24 months showed that the intervention reduced caries^9^; influenced secondary outcomes, such as energy intake from discretionary foods; and increased fruit intake,^7^ although effect sizes were small. After the collection of data at age 24 months, the intervention was offered to the (former) control group, and both groups were again followed up at age 36 months. The trial design allowed for an investigation of whether an intervention delivered from pregnancy to 18 months (immediate intervention group) led to fewer caries and better diets and anthropometric outcomes than a delayed intervention delivered from age 24 to 36 months (delayed intervention group). This design was intended to provide evidence for when to intervene.

In this secondary analysis of the Baby Teeth Talk trial, we aimed to compare the outcomes of dietary intake, anthropometric, and blood pressure measurements between children at age 36 months in the immediate intervention group and those in the delayed intervention group. Outcome data were collected from November 1, 2014, to February 28, 2016, in participant homes or public locations.

## Methods

Approval to conduct the trial was granted by the Aboriginal Health Council of South Australia and the University of Adelaide. Written informed consent was obtained from participating women. We followed the Consolidated Standards of Reporting Trials (CONSORT) reporting guideline.

The Baby Teeth Talk trial was a statewide single-blind, 2-group RCT that was conducted across the state of South Australia, Australia, involving Aboriginal and/or Torres Strait Islander children and their carers (caregivers). The collection of outcome data at age 36 months was planned from trial conceptualization (see the statistical analysis plan in Supplement 1). However, the addition of diet and anthropometric data occurred during the pregnancy recruitment phase when funding and expertise became available.

### Participants, Randomization, and Interventions

Women who self-identified as being Aboriginal and/or Torres Strait Islander, reported being pregnant with an Aboriginal and/or Torres Strait Islander baby, or delivered in the previous 6 weeks were eligible to participate. Questions on self-reported Aboriginal and/or Torres Strait Islander status were based on items used in standard Australian government surveys. Participants were recruited at public hospital antenatal clinics, at Aboriginal Community Controlled Health Organizations, and through word of mouth. Enrollment occurred between February 1, 2010, and May 31, 2011.

Participants were randomized (1:1 ratio) to the immediate intervention group or delayed intervention group through a central randomization service according to a schedule prepared by an independent statistician who was not part of the trial. The schedule was stratified according to 6 maternity hospitals and had random block sizes of 4, 6, and 8.

As described in the trial protocol,^12^ the immediate intervention group received the intervention during pregnancy and at age 6, 12, and 18 months, whereas the delayed intervention group received the intervention at age 24, 30, and 36 months. The intervention comprised 4 components: (1) free dental care for mothers, including help with making dentist appointments and transportation to and from appointments; (2) application of fluoride varnish to children’s teeth; (3) anticipatory guidance on oral health and dietary advice, including introducing solid foods at 6 months as well as drinking water and avoiding sugary foods and beverages at or after 6 months; and (4) motivational interviewing. Trial staff were trained in motivational interviewing, and the fidelity of motivational interviewing has been described elsewhere.^13^ The intervention was provided from February 1, 2011, to May 31, 2012.

### Outcomes at 36 Months of Age

For blinding purposes, staff who collected the outcome data were not involved in delivering the intervention. Outcome data were collected when the children were aged 36 months. Caregivers completed a short (17 items), validated food frequency questionnaire (FFQ), which was developed in Australia with children aged 2 to 5 years.^14^ The FFQ was chosen because it had been validated against 24-hour recalls and was brief to implement, and its development involving Australian children provided additional relevance for Australian contexts. At the follow-up when the children were 24 months of age, the dietary data were collected by 24-hour recalls, but the recalls proved too burdensome to use again, which is why the FFQ was selected for the follow-up when the children were aged 36 months. Completing the FFQ typically took 10 to 15 minutes.

Nine questions asked caregivers to estimate the usual frequency that the child consumed the following foods: fruits, vegetables, red meats (eg, beef, lamb, pork, and game), processed meats (eg, sausages, ham, and chicken nuggets), fried potatoes, potato crisps (chips) or salty snacks, fast-food or takeaway meals (eg, burgers and pizza), snack foods (eg, biscuits and cakes), and confectionaries. Questions about beverage consumption included the number of cups (1 cup = 250 mL) of plain milk, sweetened milk, soft drinks (eg, cola and cordial), diet drinks, fruit drinks (not 100% juice), and water. One question was about the type of milk typically consumed (eg, full cream, skim, or soy), and 2 behavioral questions asked how frequently breakfast was consumed and whether dinner was consumed while watching television. Children’s intake of foods was reported by caregivers using the following response options: number of servings per day, servings per week, servings per month, rarely or never, does not eat, does not know, and declined to answer. For the analysis, does not eat and rarely or never responses were coded as 0, and does not know and declined to answer were set to missing. All other response options were converted to frequency of servings per day.

Children’s height was measured to the nearest millimeter using a portable stadiometer. Children’s weight was measured to the nearest 0.1 kg using scales that were checked monthly using standard weights. Mid–upper arm circumference was measured to the nearest millimeter using a calibrated tape. Body mass index (BMI) was calculated as weight in kilograms divided by height in meters squared and converted to age- and sex- standardized *z* scores using the World Health Organization growth standards as the reference.^15^ Body mass index was further categorized into underweight, healthy, overweight, and obesity.^16,17^ Blood pressure was collected from the child’s right arm after measuring the mid–upper arm circumference to inform the selection of an appropriately sized cuff for the blood pressure monitor (Omron HEM-7211; Omron Corporation).

### Statistical Analysis

Secondary outcome analyses were performed according to a prewritten statistical analysis plan (Supplement 1), which was written after outcome data collection was completed. The main outcome was the frequency of consumption of discretionary beverages and foods, which is aligned with the purpose of the Baby Teeth Talk trial to reduce early childhood caries by decreasing cariogenic food consumption. Discretionary foods are defined in the Australian Guide to Healthy Eating.^18^ The main outcome was calculated by summing the frequency of the following discretionary food items in the FFQ: soft drinks and fruit drinks (not 100% juice) for discretionary beverages and confectionaries, processed meats (eg, sausages, ham, and chicken nuggets), fried potatoes, potato crisps or salty snacks, fast-food or takeaway meals (eg, burgers and pizza), and snack foods (eg, biscuits and cakes) for discretionary foods. Diet (sugar-free) beverages were not included.

Additional analyses included comparing the immediate intervention group with the delayed intervention group regarding their frequencies of consumption of each item in the FFQ as well as their anthropometric and blood pressure measurements. Children were dichotomized as consumers or nonconsumers of discretionary beverages, discretionary foods, diet drinks, milk, fruits, vegetables, and red meats.

Participant data were analyzed according to treatment randomization (intention to treat). Analyses were adjusted for stratification variables and data collector, with blood pressure variables also adjusted for child’s height. Although unadjusted and adjusted analyses were conducted, fully adjusted imputed data were the primary results. Unadjusted and nonimputed data were reported for transparency. Generalized linear models were used to compare the groups, with Poisson links for frequency outcomes and Gaussian links for anthropometric and blood pressure measurements. For categorical outcomes (type of milk, breakfast), a multinomial logit model was used; for dichotomous outcomes, a log-binomial model was used.

Missing data were imputed under the missing-at-random assumption. Fifty imputed data sets were created using the fully conditional specification method. Imputation models included the randomization strata and baseline characteristics (eg, mother’s age, educational level, annual income, number of children, and having a partner) and auxiliary variables (diet and anthropometric data collected at the 24-month follow-up as well as other dietary questions asked at the 36-month follow-up). Imputation models were generated separately for the immediate intervention and delayed intervention groups.

The trial sample size was calculated for the follow-up at age 24 months (overall primary outcome for the Baby Teeth Talk trial), in which 250 children (125 children per group) were estimated to be sufficient to detect a 25% difference in the prevalence of early childhood caries between the groups (α = .05; 80% power); this estimate was inflated by 35% to allow for attrition (200 children per group). For the secondary outcomes, a sample size of 340 children at age 36 months would be sufficient to detect a 5% reduction from the mean frequency of discretionary beverage and food consumption (45 events per month, which translated to 1.5 events per day) between the treatment and control groups (α = .05, 80% power). The baseline rate of 45 events per month was based on national data that Aboriginal and/or Torres Strait Islander children aged 2 to 3 years consume a median volume of 250 mL of soft drinks per day.^19^ The modest 5% difference in discretionary intake was considered realistic because dietary patterns are difficult to change.

Analyses were conducted using Stata, version 15 (StataCorp LLC). Two-sided hypothesis tests were used, with *P* < .05 considered to be statistically significant. Data were analyzed from October 5, 2018, to April 29, 2019.

## Results

A total of 448 mothers and 454 children were randomized to immediate intervention (223 mothers and 224 children [which included 1 set of twins]) and delayed intervention (225 mothers and 230 children [which included 5 sets of twins]). At the 36-month follow-up, 158 children (71%) from the immediate intervention group were followed up, whereas 172 children (75%) from the delayed intervention group underwent follow-up (Figure). At baseline, the women had a mean (SD) age of 24.9 (5.9) years, 171 (38%) lived in metropolitan areas, and 62 (14%) were employed. The children had a mean (SD) weight of 3.3 (0.6) kilograms at birth (data missing for 174 infants) and were born at a mean (SD) gestational age of 38.6 (2.1) weeks. Of these children, 205 (46%) were boys and 180 (40%) were girls; sex was not recorded for 63 children (14%).

**Figure.**
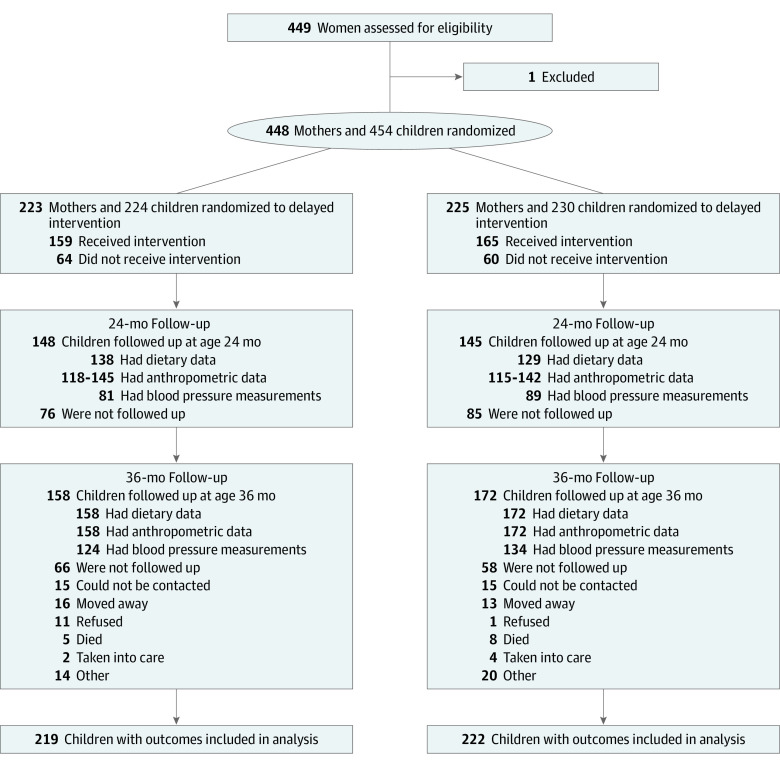
Flow of Participants Through the Trial

Through concerted efforts to contact trial participants again, we followed up more children at age 36 months (n = 330) than at age 24 months (n = 293), and the imputation methods meant that the 36-month analysis accounted for more children. Compared with adult participants who were lost to follow-up (n = 124), those who were followed up (n = 324) were more likely to live in regional areas (204 [63%] vs 67 [54%]) and be employed (55 [17%] vs 7 [6%]) (eTable 1 in Supplement 2). The characteristics of the full sample at baseline has been published elsewhere^9^; however, eTable 2 in Supplement 2 shows that the baseline characteristics of the sample at the 36-month follow-up were similar.

Dietary data in the Table show that intake of discretionary beverages by the immediate intervention group was similar to the consumption by the delayed intervention group (mean [SD]: 507 [536] mL/d vs 520 [546] mL/d; adjusted mean difference [MD], −16 [95% CI, −133 to 102] mL/d; *P* = .79). Likewise, comparisons of all other dietary outcomes were similar between the 2 groups.

**Table.** Comparison of Dietary, Anthropometry, and Blood Pressure Outcomes Between the Immediate Intervention and Delayed Intervention Groups at Age 36 Months

Outcome	Mean (SD)		Unadjusted		Adjusted	
	Immediate intervention group (n = 219)	Delayed intervention group (n = 222)	MD (95% CI)	*P* value	MD (95% CI)	*P* value
Main dietary outcomes						
Discretionary beverage, mL/d	507 (536)	520 (546)	−13 (−67 to 41)	.63	−16 (−133 to 102)	.79
Discretionary foods, servings/d	2 (2)	2 (2)	0.1 (−0.2 to 0.4)	.55	0.1 (−0.2 to 0.4)	.58
Secondary dietary outcomes						
Volume of diet drink intake, mL/d	30 (89)	41 (132)	−11 (−20 to −3)	.01	−12 (−37 to 12)	.33
Volume of milk intake, mL/d	451 (375)	397 (303)	54 (−18 to 126)	.14	47 (−26 to 120)	.21
Vegetable consumption, servings/d	2 (1)	2 (1)	0.1 (−0.1 to 0.3)	.58	0.1 (−0.2 to 0.2)	.72
Fruit consumption, servings/d	2 (1)	2 (1)	−0.1 (−0.3 to 0.2)	.61	−0.1 (−0.3 to 0.2)	.55
Volume of water intake, mL/d	949 (511)	967 (445)	−18 (−117 to 80)	.72	−11 (−116 to 94)	.84
Red meat consumption, times/d	1 (1)	1 (1)	0.1 (−0.1 to 0.2)	.33	0.1 (−0.1 to 0.1)	.52
Other eating patterns						
Breakfast consumption, times/d	1 (0.3)	1 (0.1)	0.04 (−0.02 to 0.1)	.21	0.03 (−0.03 to 0.1)	.31
Eating in front of the television, times/d	0.3 (0.4)	0.4 (0.4)	−0.1 (−0.1 to 0.1)	.67	−0.02 (−0.1 to 0.1)	.65
Anthropometry and blood pressure outcomes						
Weight *z* score	0.7 (1.0)	0.4 (1.0)	0.3 (0.1 to 0.5)	.01	0.3 (0.1 to 0.5)	.02
Height *z* score	0.0 (1.0)	−0.2 (1.0)	0.2 (−0.1 to 0.4)	.15	0.2 (−0.1 to 0.4)	.14
Arm circumference *z* score	1.6 (1.0)	1.3 (0.9)	0.3 (0.1 to 0.5)	.004	0.2 (0.1 to 0.5)	.03
BMI *z* score	1.1 (1.1)	0.9 (0.9)	0.2 (0.0 to 0.4)	.04	0.2 (0.0 to 0.4)	.04
Blood pressure, mm Hg						
Systolic	103 (20)	103 (17)	0.2 (−4 to 4)	.91	0.1 (−4 to 4)	.97
Diastolic	68 (19)	65 (15)	3 (−1 to 7)	.16	3 (−2 to 7)	.25

Abbreviations; BMI, body mass index; MD, mean difference.

Blood pressure (systolic, mean [SD], 103 [20] mm Hg vs 103 [17] mm Hg) and height (mean [SD] *z* score, 0.0 [1.0] vs –0.2 [1.0]) measurements were also similar between the 2 groups. However, the mean (SD) *z* scores of weight (0.7 [1.0] vs 0.4 [1.0]; adjusted MD, 0.3 [95% CI, 0.1-0.5]; *P* = .02), arm circumference (1.6 [1.0] vs 1.3 [0.9]; adjusted MD, 0.2 [95% CI, 0.1-0.5]; *P* = .03), and BMI (1.1 [1.1] vs 0.9 [0.9]; adjusted MD, 0.2 [95% CI, 0.0-0.4]; *P* = .04) were higher for the immediate intervention group compared with the delayed intervention group. Using the raw weight data, the adjusted MD in weight *z* scores corresponded to a difference in weight at age 36 months of about half a kilogram between children in the immediate intervention group vs those in the delayed intervention group (mean [SD], 16.0 [2.2] kg vs 15.5 [2.1] kg).

Findings from complete case comparisons were consistent with these results (eTable 3 in Supplement 2). For example, discretionary beverage intake by the immediate intervention group was similar to the intake by the delayed intervention group (mean [SD], 510 [568] mL/d vs 477 [539] mL/d; adjusted MD, −16 [95% CI, −133 to 102] mL/d). The immediate intervention and delayed intervention groups were similar in the proportion of consumers vs nonconsumers of each food category (eTable 4 in Supplement 2). For example, the proportion of children in the immediate intervention group who consumed discretionary beverages was similar to that in the delayed intervention group (92% vs 96%; adjusted risk difference, −0.04; 95% CI, −0.10 to 0.01; *P* = .15).

## Discussion

In this secondary analysis of the Baby Teeth Talk RCT involving Aboriginal and/or Torres Strait Islander children and their caregivers, the consumption of discretionary foods and beverages at age 36 months was similar between children who received the intervention from pregnancy to age 18 months (immediate intervention group) and children who received the intervention from 24 to 36 months (delayed intervention group). Blood pressure outcomes were also similar between the groups, although those in the immediate intervention group had greater *z* scores for weight, arm circumference, and body mass index than their counterparts in the delayed intervention group.

In ascertaining the best age at which to introduce an intervention, we found that the anthropometric outcomes differed from the caries finding. Intervening from 24 to 36 months resulted in better anthropometric outcomes, but as previous research has shown, intervening from pregnancy to 18 months had better caries outcomes.^9^ These novel findings present a conundrum for public health given that earlier intervention may be advantageous for caries but at the expense of poor anthropometric outcomes. Thus, recommendations could be made according to which outcome is valued more highly.

A pattern of greater *z* scores for weight, BMI, and mid–upper arm circumference emerged at the 24-month follow-up and was observed again at the 36-month follow-up. Both groups of children, in general, weighed more and therefore were unhealthier than the World Health Organization reference standard as indicated by BMI *z* scores that were approximately 1 SD higher than the reference. In addition, BMI *z* scores were higher than the World Health Organization reference for children in the immediate intervention group, suggesting that the intervention worsened BMI *z* scores. Although speculative, it is possible that the attention on food consumption in the immediate intervention group led to children consuming more food. However, increased consumption of any foods was not observed in the FFQ responses, and it would need to occur without any compensatory decrease in consumption of other foods. The extent to which social desirability bias may have played a role in reporting of children’s dietary data is unclear. Furthermore, FFQs do not directly measure energy intake and may not be sensitive enough to detect such differences in diet. Our experience in the field when collecting dietary recalls at the 24-month follow-up posed a heavy burden on participants and meant that applying a more intensive dietary assessment tool at the 36-month follow-up was not possible. A longer period may need to pass among children in the delayed intervention group before intervention-related changes in anthropometric outcomes may be observed. A key message for future studies is that oral health interventions are not benign for other aspects of children’s health, and therefore evaluations of such interventions should always extend beyond caries to other health outcomes. Longer-term follow-up of anthropometric outcomes into school age is underway and may highlight further changes.

We had difficulty finding studies for comparison of oral health interventions that tested different age ranges or specifically focused on Aboriginal and/or Torres Strait Islander families and that also collected dietary data. This scarcity was surprising considering that many oral health interventions frequently have a dietary component.^20^ Recently, Rosenstock et al^21^ showed that a 6-lesson home visiting program for Navajo mothers lowered sugar-sweetened beverage consumption among their infants and improved BMI *z* scores to 12 months of age compared with a control group that had home visits on injury prevention. The dietary intervention was designed in conjunction with Navajo communities and was delivered from 3 to 6 months by Navajo paraprofessionals.^21^ Although the intervention attempted to improve early feeding practices, contrary to expectations, a higher proportion of infants in the intervention group were introduced to complementary foods before 6 months of age, which also suggests the unintended consequences of intervening early in life.^21^ Overall, the lack of RCTs in partnership with Indigenous peoples presents opportunities for future research because trials led by Indigenous peoples are highly valued.^9,22^ Although the intervention in the Baby Teeth Talk trial was also provided to Indigenous peoples of New Zealand and Canada, there was insufficient resourcing to evaluate the effects on children’s diets or anthropometry.

We compared the diets of the children in the Baby Teeth Talk trial to national data and to studies that used the FFQ method. Compared with national data, the children in this RCT had some pronounced differences in beverage consumption.^19,23^ For example, consumption of milk, discretionary beverages, and water by the children in this trial was higher than that reported in national data, but the differences may be attributed to dietary data collection methods (national studies mostly use 24-hour recalls) or dietary recall periods and errors. Furthermore, validation of the FFQ has indicated that it is more suitable for measuring group-level intakes than absolute quantities of foods in the diet.^14,24^ The mean food intakes described in the current analysis are consistent with those in other studies that applied the FFQ to children aged 2 to 5 years.^24^ However, using the same FFQ, Xu et al^25^ reported that only 26% of children aged 24 months consumed discretionary beverages compared with more than 90% of children aged 36 months in the current study. Such marked differences in dietary intakes could be associated with high levels of social disadvantage among the study sample, but this association requires confirmation.

### Limitations

This study has limitations. Both groups received an intervention and thus comparing them with a placebo or control group was not possible. However, the purpose of comparing different intervention periods was (if successful) to inform policy and practice about the most efficacious period in childhood in which to intervene. The participant retention we achieved at the 36-month follow-up was remarkable for a hard-to-reach population with socioeconomic disadvantage. Although attrition was a concern, it appeared nondifferential by group, and we have attempted to address attrition by using multiple imputation.

## Conclusions

This secondary analysis found no substantial differences in the consumption of discretionary foods and beverages between children aged 36 months who began receiving the intervention during their mother’s pregnancy until 18 months of age and children aged 36 months who did not receive the intervention until age 24 to 36 months. However, children in the immediate intervention group had greater *z* scores for weight, arm circumference, and BMI than their counterparts in the delayed intervention group, suggesting that oral health interventions have the potential to affect anthropometric outcomes in children.

## Notes

Supplement 1. Statistical Analysis Plan

Supplement 2. **eTable 1.** Baseline Characteristics of Participants Who Were and Were Not Followed Up at Age 3 Years **eTable 2.** Baseline Characteristics of the Immediate Intervention (II) Versus Delayed (DI) Intervention Groups Who Were Followed Up to Age 3 Years **eTable 3.** Complete Case Comparison of Diet, Anthropometry and Blood Pressure Between the Immediate Intervention (II) Versus Delayed Intervention (DI) Groups at 3 Years of Age **eTable 4.** Comparison of Consumers Versus Nonconsumers of Food Categories According to Immediate Intervention (II) Versus Delayed Intervention (DI) Groups at 3 Years of Age (Imputed Outcomes)

Supplement 3. Data Sharing Statement
