# Supplementary material for: Dietary Intake and Anthropometric Measurement at Age 36 Months Among Aboriginal and/or Torres Strait Islander Children in Australia: A Secondary Analysis of the Baby Teeth Talk Randomized Clinical Trial
Source: JAMA Netw Open. 2021 Jul 8;4(7):e2114348. doi: 10.1001/jamanetworkopen.2021.14348 (PMC8267605; doi:10.1001/jamanetworkopen.2021.14348)
Supplement: Supplement 1. — Statistical Analysis Plan [file jamanetwopen-e2114348-s001.pdf]

BABY TEETH TALK (BTT) TRIAL

STATISTICAL ANALYSIS PLAN

SAP Author	Pedro Henrique Ribeiro Santiago
SAP Version	1
SAP Date	4th October 2018

Approvals

Investigators	SAP Version	Date	Signature
Pedro Henrique Ribeiro Santiago	1	22 / 08 / 2018	
A/Prof Lisa Smithers	1	4 / 10 / 2018	
Prof Lisa Jamieson	1	22 / 08 / 2018	

TABLE OF CONTENTS

1. ABBREVIATIONS 4

2. PREFACE..5

3. PURPOSE OF SAP 5

4. STUDY AIMS, HYPOTHESES AND OUTCOMES 5

4.1 Aims 5
4.2 Hypotheses..5
4.3 Outcome Variables...6

5. STUDY METHODS 6

5.1 Overall Study Design and Intervention Details 6
5.2 Funding 7
5.3 International connections 7
5.4 Ethical approval 7
5.5 Trial registration & protocol 7
5.6 Recruitment, Inclusion, Exclusion 8
5.7 Method of Treatment Assignment and Randomisation 8
5.8 Concealment of Allocation 8
5.9 Treatment Masking (Blinding) 8

6. SEQUENCE OF PLANNED ANALYSES 9

6.1 Interim Analyses 9
6.2 Final Analyses and Reporting..9

7. SAMPLE SIZE DETERMINATION..9

8. GENERAL ISSUES FOR STATISTICAL ANALYSIS 10

8.1 Analysis Approach...10
8.2 Withdrawals 10
8.3 Missing Data 10
8.4 Outliers..11
8.5 Protocol Violations and Deviations 11
8.6 Data Transformations..11
8.7 Potential Confounders...11
8.8 Multiple Comparisons and Multiplicity..12

9. DESCRIPTIVE STATISTICS...12

9.1 Participants and Withdrawals 12

9.2 Baseline Characteristics 12
9.3 Post-Randomisation Descriptive Characteristics 12

10. ANALYSIS: GENERAL INFORMATION 13

11. ANALYSIS: PRIMARY OUTCOME 13

12. SECONDARY OUTCOMES 15

12.1 Vegetables, fruits, water, milk, and red meat 17
12.2 Comparison with AGHE – Healthy eating for children guidelines 16
12.1 Behavioral eating patterns 18
12.2 Anthropometry 19
12.3 Blood Pressure 20

13. REFERENCES 21

14. APPENDIX A 24

1. ABBREVIATIONS

Abbreviation	Definition
ABS	Australian Bureau of Statistics
AG	Anticipatory guidance
AGHE	Australian Guide to Healthy Eating
AHW	Aboriginal Health Worker
ANZSCO	Australian & New Zealand Standard Classification of Occupations
ARCPOH	Australian Research Centre for Population Oral Health
BMR	Basal metabolic rate
BTT	Baby Teeth Talk
CI	Confidence interval
CONSORT	Consolidated Standards of Reporting Trials
CRF	Case Report Form
EI	Energy intake
EPAQ	Eating and Physical Activity Questionnaire
FFQ	Food Frequency Questionnaire
GEE	Generalised estimating equation
HEFC	Healthy Eating for Children
ICH	International Conference on Harmonisation
IRSAD	Index of Relative Socioeconomic Advantage and Disadvantage
MAR	Missing at random
MI	Motivational interviewing
NRV	Nutrient reference values
RCT	Randomised controlled trial
SAP	Statistical analysis plan
SD	Standard deviation
TEE	Total energy expenditure
WHO	World Health Organization

2. PREFACE

This statistical analysis plan (SAP) provides details of the planned analyses for the dietary and anthropometric outcomes of the Baby Teeth Talk (BTT) trial at three years of age. The primary outcomes of the BTT trial are dental and oral health outcomes. This particular SAP focuses on the outcomes of diet and anthropometry. The following documents were reviewed in preparation of this SAP:

- BTT protocol, published in BMC Public Health (Merrick, 2012)
- BTT Case Report Form (CRF) containing the baseline questionnaire
- BTT CRF 3-year-old follow-up questionnaire
- Reliability and validity of a short FFQ for assessing the dietary habits of 2-5-year-old children (Flood et al., 2014).
- Australian Guide to Healthy Eating - Healthy eating for children
- ICH Guidance on Statistical Principles for Clinical Trials.

3. PURPOSE OF SAP

The purpose of this SAP is to outline the planned analyses that support the completion of the primary paper for dietary and anthropometric outcomes of the BTT randomised controlled trial (RCT) at three years of age.

4. STUDY AIMS, HYPOTHESES AND OUTCOMES

4.1 Aims

The primary aim is to evaluate whether the 2 years of motivational interviewing (MI) and anticipatory guidance (AG) conducted with parents of Aboriginal or Torres Strait Islander children on the treatment group led to healthier diets and anthropometric outcomes by age 3 years compared to the late intervention of 1 year of MI and AG conducted with parents of Aboriginal or Torres Strait Islander children on the control group.

4.2 Hypotheses

The primary hypothesis is that children whose mothers were randomised to receive an intervention involving 4 MI/AG sessions from pregnancy to 2 years postnatal (treatment group) will have a lower frequency of discretionary beverages and foods consumption compared to children whose mothers were randomised to receive 3 MI/AG sessions from 2 to 3 years postnatal (control group). There are two reasons for this hypothesis.

The first reason is that an early intervention will instruct mothers on how to provide healthier food choices since childbirth and, subsequently, develop in the children healthier eating behaviours from a younger age. It is expected that the children's healthier eating behaviours will remain stable until age 3, one year after the end of the intervention, and will result in lower consumption of discretionary beverages and foods when compared to the control group. The second reason is that the treatment group received one additional MI/AG sessions compared to the control group. Therefore, it is expected that the extra behavioural guidance received by the mothers on the treatment group will result in less sugar consumption by the children at age 3 years when compared to the control group.

The secondary hypothesis is that children whose mothers were randomised to the treatment group will have an overall dietary intake closer to Australian Guide to Healthy Eating (AGHE) food and nutrition recommendations for children at age 3 years compared to the control group. Specifically, children in the treatment group will have higher intakes of vegetables, fruits, water and milk, and lower intakes of red meat than the control group. It is also hypothesized that children in the treatment group have breakfast more frequently, have dinner less frequently in front of the television and have healthier anthropometric outcomes. Healthier anthropometric outcomes include lower systolic and diastolic blood pressure; and no difference in linear growth (indicated by height-for-age z-scores) between the treatment and control group children, but fewer treatment group children with weight-for-height and weight-for-age z-scores in the overweight/obese range.

4.3 Outcome Variables

The primary outcome is the frequency of discretionary beverages and foods intake. Discretionary foods are described in the AGHE. Dietary intake was measured using a Food Frequency Questionnaire (FFQ). The 17-item FFQ assess dietary habits of young children and has been validated for children aged 2-5 years in Australia (Flood et al., 2014) (Appendix A). All primary and secondary outcomes are defined in sections 11 and 12.

5. STUDY METHODS

5.1 Overall Study Design and Intervention Details

The BTT trial is an ongoing single-blind RCT involving parents (predominantly mothers but occasionally involves relatives or other carers) of Aboriginal and/or Torres Strait Islander children. Mothers were recruited from across the entire state of South Australia and parents were enrolled during pregnancy or within the first 6 weeks postnatal. The follow-ups occurred at 2 years postpartum, 3 years postpartum and a current follow-up is taking place at 5 years postpartum.

Intervention: The intervention was developed in partnership with local Aboriginal communities and was endorsed by an Aboriginal Reference Group.

The intervention was delivered to the treatment group by an Aboriginal Health Worker (AHW) during pregnancy or the early postnatal period, and again when the child was aged 6, 12, and 18 months postpartum. The intervention included dental and health care, 4 MI/AG sessions, specifically regarding oral health and healthy foods; dental treatments for families and fluoride varnish for children. AHW staff underwent formal MI training and the fidelity of the intervention was conducted by an independent member of the Motivational Interviewing Network of Trainers. MI involves parents learning about caring for baby's teeth and learning about healthy and age-appropriate foods and drinks for their children. AG involved explaining to parents what to expect as their child grows and how to manage health-related behaviours. The control group did not receive any specific advice until their child reached 2 years of age. At 2 years age, the control group received the delayed intervention at 24, 30 and 36 months postpartum, which consisted of dental and health care, fluoride varnish for the children and 3 MI/AG with advice targeted specifically for this age group. The delaying of the intervention for the control group addresses the ethical concern that these children would receive no benefit from participation.

5.2 Funding

The BTT RCT was funded by the National Health & Medical Research Council (NHMRC) International grant (#627350). This funding also supported similar oral health interventions developed by First Nation peoples of Canada and New Zealand. The collection of diet and anthropometric data was partly funded by a NHMRC Program grant (#631947).

5.3 International connections

This SAP applies only to the SA site. None of the international partners had the facility, expertise or funding to collect and analyse dietary data from the RCTs conducted within their Indigenous communities.

5.4 Ethical approval

Ethical approval for the RCT was provided by the following ethics committees

- Aboriginal Health Council of South Australia Ethics Application number (04-09-362)
- University of Adelaide Human Research Ethics committee (H-057-2010)

5.5 Trial registration & protocol

The trial was prospectively registered with the Australian & New Zealand Clinical Trial Registry (www.anzctr.org.au) in December 2010, prior to recruitment of participants.

Registration number: ACTRN12611000111976.

5.6 *Recruitment, Inclusion, Exclusion*

Participants were recruited from hospital antenatal clinics throughout South Australia. Hospitals in metropolitan areas included the Women's & Children's Hospital, Flinders Medical Centre and the Lyell McEwin. Hospitals in non-metropolitan areas included Whylla, Port Pirie, Port Augusta, Murray Bridge, Mount Gambier, Port Lincoln, and Ceduna. Participants were also recruited through word-of-mouth, Aboriginal Community Controlled Health Organisations and community centres.

Inclusion criteria were women who were South Australian residents, either pregnant or had an Aboriginal or Torres Strait Islander child in the previous 6 weeks, and able to give informed consent.

Six staff members approached and then enrolled eligible participants using a standard script.

5.7 *Method of Treatment Assignment and Randomisation*

The randomisation schedule was prepared by a statistician at the Australian Research Centre for Population Oral Health (ARCPOH) who was not involved in the trial, using a random number generator. Participants were randomly assigned on a 1:1 basis to either the intervention or control group. Block sizes of 4, 6 and 8 were used. The size of the blocks were drawn at random and treatment allocations were balanced to ensure equal numbers of participants in each intervention arm within the blocks. The randomisation schedule was stratified by recruitment site; 1) Women's & Children's Hospital, 2) Flinders Medical Centre and Southern metropolitan areas, 3) Lyell McEwin and Northern metropolitan areas, 4) Whyalla and Port Pirie, 5) Port Augusta, 6) all other non-metropolitan areas (e.g. Murray Bridge, Mount Gambier, Port Lincoln and Ceduna).

5.8 *Concealment of Allocation*

Random allocation to the intervention or control group occurred through a central randomisation service via a computer algorithm. This method of assignment protected the randomisation schedule and ensured that no staff member could know any forthcoming treatment group assignments.

5.9 *Treatment Masking (Blinding)*

It was not possible to blind the delivery of the intervention (dental treatments, MI, AG) and therefore the participants and the AHW staff who administered the intervention

could not be blinded to treatment allocation. In order to maintain blinding for the collection of outcome data, a different set of research staff collected the 3-year follow-up data. Data collected from this study was also analysed blinded. This was achieved by including an unlabelled dummy group indicator to identify group allocation in the dataset (e.g. group A and group B, not labelled as ‘treatment’ and ‘control’). At completion of the SAP, group allocation will be revealed by the statistician who holds the randomisation code.

6. SEQUENCE OF PLANNED ANALYSES

6.1 Interim Analyses

There are no interim analyses planned for this study.

6.2 Final Analyses and Reporting

No statistical analyses will be performed until the final version of this SAP has been approved. Results of the statistical analyses will be made available to the Chief Investigators. Any post-hoc, exploratory analyses which were not identified in this SAP but are completed to support the proposed analyses will be clearly identified as exploratory. Any deviations from the analyses detailed in this SAP will be clearly documented in any reports or publications of this work.

7. SAMPLE SIZE DETERMINATION

Primary study: Sample size estimates were based on the primary outcome, early childhood caries (ECC) at 2 years of age. It was estimated that a sample size of 250 (125 per group) would be sufficient to detect at least a 25% difference in ECC prevalence between the groups with alpha 0.05 and 80% power. The 25% prevalence was based on an ECC intervention in children residing in the Northern Territory (Slade, 2011) and a motivational interviewing intervention for Indigenous children from South Asian communities (Harrison, 2007). The sample size estimates were inflated to allow for an attrition rate of at least 35% after 3 years, resulting in a target sample size of 400 participants (200 per group).

For the diet outcomes: A sample size of 340 children at 3 years of age will enable the detection of a 5% reduction in the mean of discretionary beverages and foods intake (events/month) between the treatment and control groups assuming a baseline rate of 45 events/month with 80% power (alpha 0.05, two-sided). The baseline rate of 45 events/month of discretionary beverages and foods intake is plausible since considering only sweetened beverage intake, Aboriginal and Torres Strait Islander children aged 2 to 3 years consume a median volume of 250ml (a small can) per day (ABS, 2016). This

modest 5% difference in discretionary beverages and foods intake is considered realistic given that dietary patterns are difficult to change.

8. GENERAL ISSUES FOR STATISTICAL ANALYSIS

8.1 Analysis Approach

The planned analyses will be performed using intention-to-treat principles; that participants will be analysed according to the treatment to which they were randomised.

8.2 Withdrawals

Participants who withdrew from the trial but gave permission for the use of their data will be included in the analyses. Participants who withdrew from the trial but did not give permission for the use of the data will not be included in the analyses.

8.3 Missing Data

This section is written assuming that there will be missing data and that the missing data mechanism is 'missing at random' (MAR). Multiple imputation will be undertaken to account for missing data. Fifty complete datasets will be created for analysis. Imputation will be performed within treatment group using the fully conditional specification method (also known as chained equations). Imputation models will include a range of variables including sampling design variables and baseline characteristics (e.g. maternal age, education, income, number of children, having a partner, the number of people living in the family).

To predict missingness on the primary outcome variable, three relevant auxiliary variables will be included in the imputation model: Firstly, the results of the Eating and Physical Activity Questionnaire (EPQA), a complementary FFQ applied at the 3 years follow-up composed of items with similar content. For example, the 17-item FFQ has the item "How many servings of vegetables does your child usually eat every day?", while the EPQA has the item "Yesterday, how many servings of vegetables (cooked, raw or baked beans) did your child have?". Secondly, the outcomes of the three 24-hour dietary recalls collected when children were 2 years of age. It is expected that the children eating behaviours at age 2 can help predict missingness of the children eating behaviours at age 3. Thirdly, the anthropometric outcomes at age 2 are also expected to predict missingness on the primary outcome and will be included in all imputation models.

Finally, for each outcome, any covariables that were pre-specified in the analysis will be included in the imputation model. Analyses will be performed on both the observed and imputed data, with conclusions to be drawn based on the results of the analyses

performed on the imputed data. Sensitivity analyses may be performed using different imputation models.

8.4 Outliers

Outliers will be investigated during data collection, entry, cleaning and statistical analysis. All outliers will be checked against data recorded in the CRF. Unless confirmed as data entry errors, true outliers will be retained in the analyses but errors will be corrected accordingly.

8.5 Protocol Violations and Deviations

No participant will be excluded from the intention-to-treat analyses due to protocol violations or deviations.

8.6 Data Transformations

No data transformations are planned. The statistical analyses are based on assumptions about the distribution of the outcomes. Should these assumptions turn out to be invalid, appropriate data transformations may be required. Data transformations are not planned to correct for departures from normality since the sample size is sufficient for the central limit theorem to apply (Lumley et al., 2002).

8.7 Potential Confounders

Unadjusted and adjusted analyses will be performed. The adjusted results will be used to draw conclusions about the effect of the intervention on the outcomes of interest, with unadjusted analyses performed for completeness. Adjustments will be made for stratification variables and potential confounders. Adjustment will also be made for additional baseline variables that are potential confounders for some outcomes, and in each case, adjustment variables will be specified *a priori* in this SAP. For example, child's height will be included as a confounder in analyses involving blood pressure.

If convergence is a problem, some potential confounders may need to be excluded from the adjusted analyses. Any deviation from the planned adjustment will be clearly identified.

The decision to draw conclusions from the adjusted analysis was based on the following factors. Firstly, the CPMP (2004) state that stratification variables should generally be adjusted for in the primary analysis, regardless of their effect on the outcome. We, therefore, chose to control for stratification variables.

No treatment by covariable interactions are planned.

8.8 Multiple Comparisons and Multiplicity

Multiple hypothesis tests will need to be performed as part of this SAP. No adjustment will be made for the number of analyses performed.

9. DESCRIPTIVE STATISTICS

9.1 Participants and Withdrawals

Information on participation, withdrawal, lost-to-follow-up and excluded from analyses will be reported according to the Consolidated Standards for Reporting Trials (CONSORT) statement (Moher, 2010).

9.2 Baseline Characteristics

The baseline characteristics of trial participants will be presented in table form, according to randomised group. For continuous variables, data will be reported as means and standard deviations (SD), or medians and interquartile ranges, depending on the distribution. For categorical data, number and percentages will be reported. In accordance with our ethical responsibilities and upholding confidentiality, cells with 10 or fewer participants will be suppressed. Adjacent cells will also be suppressed to avoid back calculation. No statistical comparisons of baseline characteristics will be undertaken, as any differences at baseline are due to chance (by the nature of random assignment to group).

Baseline characteristics include: maternal age, maternal Aboriginal and/or Torres Strait Islander ethnicity, location (metropolitan versus regional/remote), quintile of the Index of Relative Socioeconomic Disadvantage (IRSD) calculated from postcode (ABS, 2008), maternal education, employment (employed, government supported and other, and coded according to the Australian & New Zealand Standard Classification of Occupations (ABS, 1986)), holder of a health care card (yes, no), place of child's birth (Women's & Children's Hospital, Flinders Medical Centre & southern metropolitan area, Lyell McEwin and northern metropolitan area, Whylla & Port Pirie, Port Augusta, non-metropolitan regions), parity (0, 1, ≥2), living with a partner (yes, no), number of people >16 years living in the family home (1, 2, 3, ≥4), maternal smoking during pregnancy (never, former, current), maternal alcohol use (never, former, current), gestational age at birth, weight at birth, breastfeeding at birth (yes, no).

The characteristics of participants who were followed up at age 3 years will be compared with the participants who were not followed up (i.e. withdrawn, lost to follow up).

9.3 Post-Randomisation Descriptive Characteristics

A descriptive analysis of the post-randomisation variables listed below will be performed to compare the characteristics of participants in each treatment group. Means and SD, or medians and interquartile ranges will be reported for continuous variables. Frequencies and percentages will be reported for categorical variables.

Groups will be compared using t-tests for continuous outcomes and Chi-square tests for categorical outcomes. Post-randomisation variables to be compared are child's age at follow up and parents' characteristics, such as who is the primary carer for the child, if the mother gave birth to other children after the beginning of the study, if parents care for other children and how many, employment status and relationship status.

10. ANALYSIS: GENERAL INFORMATION

In this section, general information regarding the analysis that will be applied to all primary and secondary outcomes are described.

Outcome: A detailed description of the outcome variable, including the type of variable, and how it will be calculated (if applicable).

Effect: The measure of treatment effect to be calculated.

Analysis: The type of statistical analysis to be performed.

Adjustments: The baseline covariables (stratification variables and potential confounders) to include in the adjusted analysis.

Additional comments for binary outcomes: If the number of participants experiencing the outcome is considered too small for the planned analysis to be sensible, a Fisher's exact test will be performed instead with no adjustment made for baseline covariates. For analyses performed using a log-binomial model, a log Poisson model with robust variance estimation will be used if the model fails to converge.

11. ANALYSIS: PRIMARY OUTCOME

The primary outcome is the frequency of discretionary beverages and foods intake. Dietary intake was measured using a 17-item FFQ validated for children aged 2-5 years in Australia (Flood et al., 2014). One additional question "How many cups of flavoured milk does your child usually drink in a day? Includes milo, nesquick, ovomaltine" was included in the questionnaire. The reason for the inclusion was to distinguish the intake of non-cariogenic sugars (e.g. lactose) in regular milk compared to added sugars in flavoured milk. Since the higher proportion of free sugars consumption by children in Australia comes from discretionary beverage and foods, the frequency of discretionary

beverages and foods intake was chosen as a proxy variable for free sugar intake by the children. The study primary outcome is aligned with the intention of the BTT to reduce early childhood caries and reducing sugar intake was a specific component of the dietary advice given as part of the intervention. The details of the calculation of the frequency of discretionary beverages and foods intake using the 17-item FFQ are displayed below.

Frequency calculation: The FFQ 17 items have the categories of “_ servings per day”, “ _ servings per week”, “_ servings per month”, “rarely/never”, “doesn’t eat”, “don’t know” and “refused”, and the participant could respond to only one of them. The frequency of discretionary beverages and foods intake will be converted to the number of occasions per month for analysis. It is assumed that average consumption is constant over the month. Responses given as “rarely/never” will be coded as zero under the assumption that rare occasions are inconsequential to overall sugar intake. In case of responses to the “doesn’t eat” category, the number of events will be zero. In case of responses to the “don’t know” or “refused” category, the number of events will be set to missing and imputed.

Units: In the 17-item FFQ, items regarding beverage intake contain responses that evaluate the number of *cups* (e.g. “ _ cups per month”), while items regarding discretionary food intake contain responses that evaluate the number of *times* (e.g. “ _ times per month”). The frequency of discretionary food intake will have the unit of times/month. Since in the 17-item FFQ questionnaire it is specified that 1 cup = 250ml, the frequency of beverage intake will be converted to the unit of ml/month. Therefore, the answer to these questions also inform the amount of beverage intake. Items that measure vegetables and fruit intake evaluate the number of *servings* (e.g. “_ servings per month”) and the frequency will be measured as servings/month.

Intake calculation: The overall discretionary beverage intake will be calculated by summing the score of the three items: “How many cups of flavoured milk does your child usually drink in a day? Includes milo, nesquick, ovaltine”, “How many cups of soft drink, cordials or sports drink, such as lemonade or Gatorade does your child usually drink?”, and “How many cups of fruit juice does your child usually drink?”. Since in this study beverage intake is used a proxy for free sugar consumption, the summated score will not include the item “How many cups of diet soft drink or diet cordial such as Diet Coke or Diet Sprite or Coke Zero does your child usually drink?” which measures the consumption of sugar-free drinks. This item will be analysed separately. Therefore, the beverage intake by type of beverage will be calculated by using the score of the four items independently.

The overall discretionary food intake will be calculated by summing the score of the six items: “How often does your child eat meat products such as sausages, frankfurters, fritz, ham, hamburgers or chicken nuggets?”, “How often does your child eat hot chips, French fries, wedges or fried potatoes?, “How often does your child eat potato crisps or other salty snacks (such as Twisties or corn chips)?”, “How often does your child have meals or snacks such as burgers, pizza, chicken or chips from places like local take-

away shops, McDonalds, Hungry Jacks, Pizza Hut, KFC or Red Rooster?", "How often does your child have snack foods such as sweet or savoury biscuits, cakes, doughnuts or muesli bars?" and "How often your child eat confectionery, such as lollies and chocolate?". The discretionary food intake by type of food will be calculated by using the score of the six items independently.

Measurement assumptions: The FFQ items were used to calculate the frequency of discretionary beverage and food intake as a proxy for free sugar consumption. For all items measuring frequency of consumption, we make no assumptions about the volume or portion of food consumed at each occasion. In this study, the frequency of discretionary beverages and foods intake will not be converted into nutrients to avoid the inclusion of further measurement assumptions.

The dietary database will be developed in wide form, with a single row per participant.

Outcome:	Volume of discretionary beverage intake (ml/month) (Distributional assumption: the volume of beverage intake follows a Normal distribution)<br><br>Frequency of discretionary foods intake (servings/month) (Distributional assumption: the frequency of discretionary foods intake follows a Poisson distribution)
Effect:	*For discretionary beverages:* Mean difference of discretionary beverages intake between treatment and control group.<br><br>*For discretionary foods:* Mean difference in the frequency of discretionary food intake between treatment and control group.
Analysis:	A generalised linear model with Gaussian family and identity function that includes group allocation, adjustment for stratification variables (i.e. centre) and staff member who applied the questionnaire.<br><br>A generalised linear model with Poisson family and identity function with robust standard errors that includes group allocation, adjustment for stratification variables (i.e. centre) and staff member who applied the questionnaire.

12. SECONDARY OUTCOMES

The secondary outcomes are: the frequency of vegetables, fruits, water, milk and red meat; type of milk most frequently consumed; frequency of having breakfast and eating dinner in front of the televion; and anthropometric outcomes. Except for the anthropometric outcomes, the secondary outcomes will be measured with the 17-item FFQ (Appendix A) and the considerations of Section 11 (i.e. frequency calculation, units, intake calculation, and measurement assumptions) apply.

12.1 Comparison with AGHE – Healthy eating for children guidelines

The AGHE – Healthy eating for children (HEFC) provides guidelines about the kinds of food and amount that children need to eat for their health and well-being. All recommendations are based on scientific evidence. The HEFC provides recommendations on serves per day for vegetables, fruits, and milk.

Outcomes (units):	• Vegetables (servings/month)<br>• Fruits (servings/month)<br>(Distributional assumption: frequencies follow a Poisson distribution)<br><br>• Milk (ml/month)<br>(Distributional assumption: the volume follows a Normal distribution)
Effect:	Unadjusted mean differences in the frequency of vegetables, fruit and milk intake between treatment and control group.<br><br>Adjusted mean differences in the frequency of vegetables and fruit intake between treatment and control group.<br><br>Adjusted mean difference of milk intake between treatment and control group.
Analysis:	An unadjusted generalised linear model with Poisson family and identity function with robust standard errors that includes group allocation comparing mean frequency of vegetables, fruit and milk intake by the children on the treatment and control group.<br><br>A generalised linear model with Poisson family and identity function with robust standard errors that includes group allocation, adjustment for stratification variables (i.e. centre) and staff member who applied the questionnaire.

	A generalised linear model with Gaussian family and identity function that includes group allocation, adjustment for stratification variables (i.e. centre) and staff member who applied the questionnaire.

Additionally, the HEFC recommended serves will be multiplied by thirty and compared with the monthly serves of vegetables, fruits and milk for treatment and control group. The comparison with the HEFC is possible since the items regarding vegetables, fruit and milk on the 17-item FFQ display standard sizes (i.e. one serving=1/2 cup of cooked vegetables or 1 cup salad vegetables; one serving=one medium piece or two small pieces of fruit; and 1 cup=250mL).

12.2 Water, red meat, and type of milk consumption

Firstly, the treatment and control group will be compared regarding the volume of water and frequency of red meat intake. Secondly, the type of milk consumed will be evaluated through the item: “What type of milk does your child usually consume?”, which has the categories of “Whole milk (regular, full cream)”, “Flavoured milk”, “Reduced fat milk”, “Skimmed milk”, “Regular soya milk”, “Reduced fat soya milk”, “Evaporated or sweetened condensed” and “Other”. The categories of “Reduced fat milk” and “Skimmed milk” will be combined and used as a reference group. The category of “Reduced fat soya milk” will also be combined with the previous two in case of low frequency of responses by the children.

Outcomes (units):	• Water (cups/month)<br>(Distributional assumption: the volume follow a Normal distribution)<br><br>• Red meat (times/month)<br>(Distributional assumption: frequencies follow a Poisson distribution)
Effect:	Mean difference of the water intake between treatment and control group.<br><br>Mean difference of the red meat intake between treatment and control group.<br><br>For pattern of milk consumption: the relative risk ratio (RRR) of consuming a particular type of milk in the treatment compared with control group.

Analysis:	A generalised linear model with Gaussian family and identity function that includes group allocation, adjustment for stratification variables (i.e. centre) and staff member who applied the questionnaire.<br><br>A generalised linear model with Poisson family and identity function with robust standard errors that includes group allocation, adjustment for stratification variables (i.e. centre) and staff member who applied the questionnaire.<br><br>A multinomial logit model that includes group allocation, adjustment for stratification variables (i.e. centre) and staff member who applied the questionnaire.

12.3 Consumers vs non-consumers

An indicator variable will be created to categorise children into consumes (or non-consumers) of discretionary beverages, discretionary foods, vegetables, fruits, milk, and red meat accordingly to their responses on the FFQ.

Outcomes (units):	Consumers/non-consumers is dichotomised (0=non-consumer, 1=consumer) (Distributional assumption: frequencies follow a binomial distribution)
Effect:	For consumers of food groups: the risk difference (RD) of consuming food from a particular food group in the treatment compared with control group.
Analysis:	A generalised linear model with binomial family and identity function model with robust errors will be used for consumers versus non-consumers outcomes.

12.1 Additional eating patterns

Two mechanisms have been proposed to link television viewing and obesity: reduced energy expenditure from lack of physical activity and increased food intake, either during viewing or from being exposed to food advertisement (Jordan, 2010; Robinson, 1998). In addition, children who eat breakfast on a consistent basis tend to have

superior nutritional profiles than those who regularly skip breakfast (Rampersaud et al., 2005). The treatment and control group children will be compared regarding the frequency of eating breakfast (times/month) and eating dinner in front of the television.

Outcomes (units):	• Frequency of eating breakfast (times/month)<br>• Frequency of eating dinner in front of the television (times/month)<br>(Distributional assumption: frequencies follow a Poisson distribution)
Effect:	Mean difference of the frequency between treatment and control group.
Analysis:	A generalised linear model with Poisson family and identity function with robust standard errors that includes group allocation, adjustment for stratification variables (i.e. centre) and staff member who applied the questionnaire.

12.2 Anthropometry

Children's weight, height and mid-upper arm circumference are measured at the 3-year follow-up by trained research staff according to standard procedures (de Onis et al 2004; WHO,2008) and a written protocol (BTT protocol). Duplicate measures were taken and the average of the duplicates will be used for these analyses.

Anthropometric measurements will be converted to the age- and sex-adjusted z-scores; weight-for-age (WfA), height-for-age (HfA), body mass index for age (BMIz) and arm circumference for age (AfA). BMI is calculated from weight (in kg) divided by height^2 (in metres^2). The World Health Organization child growth standards (WHO, 2006) will be used as the reference. Z-scores will be calculated using the zanthro program in Stata.

Outcomes:	• WfA<br>• HfA<br>• BMIz<br>• AfA<br>(Assumption: normal distribution)
Effect:	Mean difference in z-scores (treatment minus control)
Analysis:	A generalised linear model with adjustment for stratification variables (i.e. centre).

In addition, BMIz scores will be categorised into the following categories; thin, healthy weight (reference), overweight, and obese, using International Obesity Taskforce values for children (Cole, 2000, 2007). If there are too few children in each category, the thin and healthy groups (and the overweight and obese groups) may be combined.

Outcomes:	BMI categories (healthy weight as the referent category)
Effect:	The RD of being in a BMI category relative to the healthy weight category in the treatment group compared with control group.
Analysis:	A generalised linear model with binomial family and identity function model with robust errors, with adjustment for stratification variables (i.e. centre).

12.3 Blood Pressure

A recent paper indicates that added sugars are associated with an increase of diastolic blood pressure in children (Kell et al., 2014). Blood pressure was measured in duplicate using a portable oscillometric blood pressure monitor with an appropriately-sized pediatric cuff, according to a standard protocol (Pickering 2005; BTT protocol).

Outcome:	Blood pressure (mm Hg)<br>(Assumption: normal distribution)
Effect:	Difference in mean blood pressure (treatment minus control)
Analysis:	A generalised linear model with adjustment for stratification variables (i.e. centre), child’s height and weight.

13. REFERENCES

ABS. Australian Standard Classification of Occupations (ASCO) statistical classification. 1st edition. Canberra, Australia: Commonwealth of Australia, 1986.

ABS. Information paper: an introduction to socio-economic for areas (SEIFA). Canberra, Australia: Commonwealth of Australia, 2008.

ABS. Australian Health Survey: Nutrition First Results – Food and Nutrients, 2011-2012. Catalogue number 464.0.55.007. Canberra, Australia: Commonwealth of Australia, 2014.

ABS. Australian Health Survey: Consumption of added sugars, 2011-2012. Canberra, Australia: Commonwealth of Australia, 2016.

Black AE. Critcial evaluation of energy intake using the Goldberg cut-offs for energy intake: basal metabolic rate. A practical guide to its calculation, use and limitations. *Int J Obes Relat Metab Disord* 2000; 24: 119-1130.

Cole TJ, Bellizzi MC, Flegal KM, Diet WH. Establishing a standard definition for child overweight and ovesity worldwide: international survey. *BMJ* 2000; 335: 194.

Cole TJ, Felgal KM, Nicholls D, Jackson AA. Body mass index cut offs to define thinness in children and adolescents: international survey. *BMJ* 2007; 335:194.

Committee for Proprietary Medicinal Products (CPMP). Points to consider on adjustment for baseline covariates. *Statistics in Medicine* 2004; 23(5):701-709.

de Onis M, Onyango A, Van den Broek J et al. Measurement and standardization protocols for anthropmetry used in the construction of a new international growth reference. *Food Nutrition Bulletin* 2004; 24:1.

Department for Health and Ageing. Australian National Children's Nutrition and Physcial Activity Survey: User Guide. Canberra, Australia: Commonwealth of Australia, 2007.

Flood, V. M., Wen, L. M., Hardy, L. L., Rissel, C., Simpson, J. M., & Baur, L. A. (2014). Reliability and validity of a short FFQ for assessing the dietary habits of 2–5-year-old children, Sydney, Australia. *Public Health Nutrition, 17*(3), 498-509.

Goldberg GR, Black AE, Jebb SA, Cole TJ, Murgatroyd PR, Coward WA, Prentice AM. Critical evaluation of energy intake data using fundamental principles of energy physiology: 1. Derivation of cut-off limits to identify under-recording. *European Journal of Clinical Nutrition* 1991; 45:569-581.

Graham JW, Olchowski AE, Gilreath TD. How many imputations are really needed? Some practical clarifications of multiple imputation theory. *Prevention Science* 2007; 8:206-213.

Harrison R, Benton T, Everson-Stewart, Weinstein P. Effect of motivational interviewing on rates of early childhood careis: a randomized trial. *Pediatr Dent* 2007; 29(1): 16-22.

Jordan, A. B. (2010). Children’s television viewing and childhood obesity. *Pediatric Annals, 39*(9), 569-573.

Kell, K. P., Cardel, M. I., Bohan Brown, M. M., & Fernández, J. R. (2014). Added sugars in the diet are positively associated with diastolic blood pressure and triglycerides in children–. *The American journal of clinical nutrition, 100*(1), 46-52.

Lumley T, Diehr P, Emerson S, Chen L. The importance of the normality assumption in large public health data sets. *Annual Review of Public Health* 2002; 23:151-169.

Merrick J, Chong A, Parker E, Roberts-Thomson K, Misan G, Spencer J, Broughton J, Lawrence H, Jamieson L. Reducing disease burden and health inequalities arising from chronic disease among Indigenous children: an early childhood caries intervention. *BMC Public Health* 12:323.

Moher D, Hopewell S, Schulz KF, Montori V, Gotzsche PC, Devereaux PJ, Elbourne D, Egger M, Altman DG. CONSORT 2010 Explanation and elaboration: updated guidelines for reporting parallel group randomised trials. *BMJ* 2010; 340;c869.

Pickering TG, Hall JE, Appel LJ et al. Recommendations for blood pressure measurement in humans and experimental animals: Part 1: blood pressure measurement in humans: a statement for professionals from the subcommittee of professional and public education for the American Heart Association Council on high blood pressure research. *Circulation* 2005; 111: 697-716.

Rampersaud, G. C., Pereira, M. A., Girard, B. L., Adams, J., & Metzl, J. D. (2005). Breakfast habits, nutritional status, body weight, and academic performance in children and adolescents. *Journal of the American Dietetic Association, 105*(5), 743-760.

Rangan AM, Flood VM, Gill TP. Misreporting of energy intake in the 2007 Australian children’s survey: identification, characteristics and impact of misreporters. *Nutrients* 2011; 3(2): 186-199. Doi: 10.3390/nu3020186.

Robinson, T. N. (1998). Does television cause childhood obesity? *JAMA, 279*(12), 959-960.

Schofield WN. Predicting basal metabolic rate, new standards and review of previous work. *Hum Nutr Clin Nutr* 1985; 39c(Suppl 1):5-41.

Schulz KF, Grimes DA. Multiplicity in randomised trials I: endpoints and treatments. *Lancet* 2005; 365:1348-1353.

Slade GD, Bailie RS, Roberts-Thomson K, Leach AJ, Raye I, Endean C, Simmons B, Morris P. Effect of health promotion and fluoride varnish on dental caries among Australian Aboriginal children: results from a community-randomized controlled trial. *Community Dent Oral Epidemiol* 2011; 39:29-43.

Webb K, Lahti-Koski M, Rutishauser I, Hector DJ, Knezevic N, Gill T, Peat, JK, Leeder SR; CAPS team. *Public Health Nutrition* 2006; 9(8): 1035-1044.

WHO. WHO child growth standards: training course on child growth assessment. Geneva, Switzerland, 2008

WHO Multicentre Growth Reference Study Group. WHO Child Growth Standards based on length/height, weight and age. *Acta Paediatrica Supplement* 2006; 450:76-85.

WHO. Anthro for personal computers, version 3, 2009: Software for assessing growth and development of the world's children. Geneva: WHO, 2009.

14. APPENDIX A

Appendix

Young Children's Food and Drink Study

Thank you for agreeing to participate in this study about short nutrition questions of young children. The following set of short questions will take approximately 10 to 15 min to complete. The answers are confidential and will only be seen by the survey team. No one else will have access to your information. We are interested to learn more about your pre-school child's usual eating habits. I'm going to read you a list of different food and drinks. Please tell me how much of these foods and drinks [child] usually consumes per day or per week.

The first two questions are about fruit and vegetables:

Q1. How many servings of vegetables does [child's name] usually eat each day? (one serving = 1/2 cup cooked vegetables or 1 cup of salad vegetables)
1. ______ servings per day 2. ______ servings per week 3. Doesn't eat vegetables
4. Don't know 5. Refused

Q2. How many servings of fruit does [child's name] usually eat each day? (one serving = one medium piece or two small pieces of fruit or one cup of diced pieces)
1. ______ servings per day 2. ______ servings per week 3. Doesn't eat fruit
4. Don't know 5. Refused

The next few questions ask how often your child eats some foods:

Q3. How often does [child's name] eat red meat, such as beef or lamb? Include all steaks, chops, roasts, mince, stir fries and casseroles. Do not include pork or chicken.
Longer list (do not read out): Veal, offal (liver, kidney), mutton, game (buffalo, crocodile, goanna, goat, hare, kangaroo, rabbit, snake, venison, wild boar)
1. ______ times per day 2. ______ times per week 3. ______ times per month
4. Rarely/never 5. I don't know/can't say 6. Refused

Q4. How often does [child's name] eat meat products such as sausages, frankfurters, devon, ham, hamburgers or chicken nuggets?
Longer list (do not read out): Salami, bacon, meat pies, sausage rolls, luncheon meats, delicatessen meats, meat paste, liver paste, pate, saveloys, cheerios, hot dogs, rissoles, canned meats, smoked chicken, other smoked meats
1. ______ times per day 2. ______ times per week 3. ______ times per month
4. Rarely/never 5. I don't know/can't say 6. Refused

Q5. How often does [child's name] eat hot chips, French fries, wedges or fried potatoes?
1. ______ times per day 2. ______ times per week 3. ______ times per month
4. Rarely/never 5. I don't know/can't say 6. Refused

Q6. How often does [child's name] eat potato crisps or other salty snacks (such as Twisties or corn chips)?
1. ______ times per day 2. ______ times per week 3. ______ times per month
4. Rarely/never 5. I don't know/can't say 6. Refused

Q7. How often does [child's name] have meals or snacks such as burgers, pizza, chicken or chips from places like McDonalds, Hungry Jacks, Pizza Hut, KFC, Red Rooster or local take-away food places?
1. _______ times per day 2. ______ times per week 3. ______ times per month
4. Rarely/never 5. I don't know/can't say 6. Refused

Q8. How often does [child's name] have snack foods such as sweet or savoury biscuits, cakes, doughnuts or muesli bars?
1. ______ times per day 2. ______ times per week 3. ______ times per month
4. Rarely/never 5. I don't know/can't say 6. Refused

Q9. How often does [child's name] eat confectionery, such as lollies and chocolate?
1. ______ times per day 2. ______ times per week 3. ______ times per month
4. Rarely/never 5. I don't know/can't say 6. Refused

Q10. How often does [child's name] usually have something for breakfast?
1. Every day 2. ______ times per week 3. ______ times per month
4. Rarely/never 5. I don't know/can't say 6. Refused

Q11. How often does [child's name] eat dinner in front of the television?
1. Every day 2. ______ times per week 3. ______ times per month
4. Rarely/never 5. I don't know/can't say 6. Refused

Appendix *Continued*

The next few questions are about the quantity of drinks your child usually consumes:

Q12. How many cups of milk does [child's name] usually drink in a day? Includes cow's milk, soya milk, milk on cereal, flavoured milks (one cup = 250 ml, a household tea cup)
1. ______ cups per day 2. ______ cups per week 3. ______ cups per month
4. Doesn't drink cow's milk or other milk 5. Don't know 6. Refused

Q13. What type of milk does [child's name] usually consume?
1. Whole milk (regular, full-cream) 2. Low – reduced fat milk 3. Skimmed milk
4. Evaporated or sweetened condensed 5. Soya milk, regular. Please specify ______________ 6. Soya milk, reduced fat. Please specify______________
7. None of the above 8. Don't know 9. Refused

Q14. How many cups of soft drink, cordials or sports drink, such as lemonade or Gatorade does [child's name] usually drink? (1 cup = 250 ml. One can of soft drink = $1\frac{1}{2}$ cups. One 500 ml bottle of Gatorade = 2 cups)
1. ______ cups per day 2. ______ cups per week 3. _______ cups per month
4. Doesn't drink soft drink 5. Don't know 6. Refused

Q15. How many cups of diet soft drink or diet cordial such as Diet Coke or Diet Sprite or Coke Zero does [child's name] usually drink? (1 cup = 250 ml. One can of soft drink = $1\frac{1}{2}$ cups. One 500 ml bottle of Gatorade = 2 cups)
1. _______ cups per day 2. ______ cups per week 3. ______ cups per month
4. Doesn't drink diet soft drink 5. Don't know 6. Refused

Q16. How many cups of fruit juice does [child's name] usually drink? (1 cup = 250 ml, a household tea cup or 1 large popper)
1. _______ cups per day 2. ______ cups per week 3. ______ cups per month
4. Doesn't drink juice 5. Don't know 6. Refused

Q17. How many cups of water does [child's name] usually drink in a day? (1 cup = 250 ml, a household tea cup, 1 average bottle of water = $2\frac{1}{2}$ cups)
1. ______ cups per day 2. ______ cups per week 3. ______ cups per month
4. Doesn't drink water 5. Don't know 6. Refused
