# Supplementary material for: Dietary Intake and Anthropometric Measurement at Age 36 Months Among Aboriginal and/or Torres Strait Islander Children in Australia: A Secondary Analysis of the Baby Teeth Talk Randomized Clinical Trial
Source: JAMA Netw Open. 2021 Jul 8;4(7):e2114348. doi: 10.1001/jamanetworkopen.2021.14348 (PMC8267605; doi:10.1001/jamanetworkopen.2021.14348)
Supplement: Supplement 2. — eTable 1. Baseline Characteristics of Participants Who Were and Were Not Followed Up at Age 3 Years eTable 2. Baseline Characteristics of the Immediate Intervention (II) Versus Delayed (DI) Intervention Groups Who Were Followed Up to Age 3 Years eTable 3. Complete Case Comparison of Diet, Anthropometry and Blood Pressure Between the Immediate Intervention (II) Versus Delayed Intervention (DI) Groups at 3 Years of Age eTable 4. Comparison of Consumers Versus Nonconsumers of Food Categories According to Immediate Intervention (II) Versus Delayed Intervention (DI) Groups at 3 Years of Age (Imputed Outcomes) [file jamanetwopen-e2114348-s002.pdf]

Supplementary Online Content

Smithers LG, Hedges J, Ribeiro Santiago PH, Jamieson LM. Dietary intake and anthropometric measurement at age 36 months among Aboriginal and/or Torres Strait Islander children in Australia: a secondary analysis of the Baby Teeth Talk randomized clinical trial. *JAMA Netw Open*. 2021;4(7):e2114348. doi:10.1001/jamanetworkopen.2021.14348

eTable 1. Baseline Characteristics of Participants Who Were and Were Not Followed Up at Age 3 Years

eTable 2. Baseline Characteristics of the Immediate Intervention (II) Versus Delayed (DI) Intervention Groups Who Were Followed Up to Age 3 Years

eTable 3. Complete Case Comparison of Diet, Anthropometry and Blood Pressure Between the Immediate Intervention (II) Versus Delayed Intervention (DI) Groups at 3 Years of Age

eTable 4. Comparison of Consumers Versus Nonconsumers of Food Categories According to Immediate Intervention (II) Versus Delayed Intervention (DI) Groups at 3 Years of Age (Imputed Outcomes)

This supplementary material has been provided by the authors to give readers additional information about their work.

© 2021 Smithers LG et al. *JAMA Network Open.*

eTable 1. Baseline Characteristics of Participants Who Were and Were Not Followed Up at Age Three

	Followed up<br>n = 324	Not followed up<br>n = 124
**Maternal age (years)**		
Mean ± SD	24.9 ± 5.8	25.0 ± 6.2
Missing	25 (8%)	6 (5%)
**Maternal Aboriginality, n(%)**		
Aboriginal, Torres Strait Islander or both	260 (80%)	102 (82%)
Other	55 (17%)	18 (15%)
Missing	9 (3%)	4 (3%)
**Location, n(%)**		
Regional	204 (63%)	67 (54%)
Metropolitan	116 (36%)	55 (44%)
Missing	4 (1%)	2 (1%)
**IRSAD, n(%)**		
1st quintile	175 (54%)	62 (50%)
2nd quintile	54 (17%)	27 (22%)
3rd quintile	74 (23%)	21 (17%)
4th quintile	10 (3%)	9 (7%)
5th quintile	5 (1%)	2 (2%)
Missing	6 (2%)	3 (2%)
**Maternal education, n(%)**		
No schooling	6 (2%)	1 (0.01%)
Primary school	5 (1%)	2 (2%)
High school	218 (67%)	84 (68%)
Trade school	66 (21%)	24 (19%)
University	22 (7%)	9 (7%)
Missing	7 (2%)	4 (3%)
**Health care card, n(%)**		
No	66 (20%)	11 (9%)
Yes	248 (76%)	103 (83%)
Missing	10 (4%)	10 (8%)
**Parity, n(%)**		
Mean ± SD	1.5 ± 0.6	1.5 ± 0.7
Missing	114 (35%)	37 (30%)
**Number of people living in the house, n(%)**		
Mean ± SD	1.8 ± 0.9	1.8 ± 0.7
Missing	20 (6%)	8 (6%)
**Employment, n(%)**		
Employed	55 (17%)	7 (6%)
Social welfare support	246 (76%)	104 (84%)
Other	15 (5%)	7 (6%)
Missing	8 (2%)	6 (4%)
**Car ownership, n(%)**		
Yes	171 (53%)	53 (43%)
No	145 (45%)	67 (54%)
Missing	8 (2%)	4 (3%)
**Smoking, n(%)**		
Never	79 (24%)	24 (19%)
Used to	83 (26%)	32 (26%)
Currently	154 (48%)	64 (52%)
Missing	8 (2%)	4 (3%)
**Alcohol consumption, n(%)**		

© 2021 Smithers LG et al. *JAMA Network Open.*

Never	28 (9%)	7 (6%)
Used to	255 (79%)	105 (85%)
Currently	33 (10%)	7 (6%)
Missing	8 (2%)	5 (3%)

Abbreviation: IRSAD, index of relative socioeconomic advantage and disadvantage; TSI, Torres Strait Islander

© 2021 Smithers LG et al. *JAMA Network Open.*

eTable 2. Baseline Characteristics of the Immediate Intervention (II) Versus Delayed (DI) Intervention Groups Who Were Followed Up to Age Three

	II intervention<br>n = 158	DI intervention<br>n = 172
**Maternal age (years)**		
Mean ± SD	24.9 ± 5.7	24.9 ± 5.8
Missing, n(%)	11 (7%)	17 (10%)
**Maternal Aboriginality, n(%)**		
Aboriginal, TSI, or both	131 (83%)	130 (76%)
Other	22 (14%)	36 (21%)
Missing	5 (3%)	6 (3%)
**Location, n(%)**		
Regional	106 (67%)	99 (58%)
Metropolitan	51 (32%)	67 (39%)
Missing	1 (1%)	6 (3%)
**IRSAD, n(%)**		
1st quintile, most disadvantaged	87 (55%)	90 (52%)
2nd quintile	21 (13%)	34 (20%)
3rd quintile	41 (26%)	33 (19%)
4th quintile	7 (4%)	3 (2%)
5th quintile, most advantaged	1 (1%)	4 (2%)
Missing	1 (1%)	8 (5%)
**Education, n(%)**		
No schooling	3 (2%)	3 (2%)
Primary school	2 (1%)	3 (2%)
High school	105 (67%)	114 (66%)
Trade school	32 (20%)	36 (21%)
University	13 (8%)	9 (5%)
Missing	3 (2%)	7 (4%)
**Health care card, n(%)**		
No	34 (21%)	33 (19%)
Yes	119 (75%)	131 (76%)
Missing	5 (4%)	8 (5%)
**Parity**		
Mean ± SD	1.5 ± 0.6	1.4 ± 0.7
Missing	59 (37%)	58 (34%)
**Number of people living in the house, n(%)**		
Mean ± SD	1.8 ± 0.9	1.9 ± 0.9
Missing	8 (5%)	16 (9%)
**Employment, n(%)**		
Job	30 (19%)	25 (14%)
Social welfare support	119 (75%)	130 (76%)
Other	6 (4%)	9 (5%)
Missing	3 (2%)	8 (5%)
**Car ownership, n(%)**		
Yes	80 (51%)	94 (55%)
No	74 (47%)	71 (41%)
Missing	4 (2%)	7 (4%)
**Smoking, n(%)**		
Never	38 (24%)	43 (25%)
Used to	45 (28%)	39 (23%)
Currently	71 (45%)	83 (48%)
Missing	4 (3%)	7 (4%)
**Alcohol consumption, n(%)**		

© 2021 Smithers LG et al. *JAMA Network Open.*

	II intervention<br>n = 158	DI intervention<br>n = 172
Never	14 (9%)	14 (8%)
Used to	121 (76%)	136 (79%)
Currently	19 (12%)	15 (9%)
Missing	4 (3%)	7 (4%)

Abbreviation: IRSAD, index of relative socioeconomic advantage and disadvantage; TSI, Torres Strait Islander

© 2021 Smithers LG et al. *JAMA Network Open.*

eTable 3. Complete Case Comparison of Diet, Anthropometry and Blood Pressure Between the Immediate Intervention (II) Versus Delayed Intervention (DI) Groups at Three Years of Age

	II n = 158	DI n = 172	Unadjusted			Adjusted		
	Mean ± SD	Mean	MD	95% CI	P	MD	95% CI	P
**Main dietary outcomes**								
Discretionary beverage (mL/day)	477 ± 539	510 ± 568	-33	-157, 91	0.599	-26	-154, 102	0.70
Discretionary foods (servings/day)	2 ± 2	2 ± 1	0.1	-0.2, 0.4	0.551	0.1	-0.3, 0.4	0.72
**Secondary dietary outcomes**								
Volume of diet drinks intake (mL/day)	28 ± 92	41 ± 138	-13	-38, 13	0.340	-12	-39, 15	0.39
Volume of milk intake (mL/day)	447 ± 379	399 ± 304	48	-28, 123	0.216	35	-41, 112	0.36
Vegetable consumption (servings/day)	2 ± 1	2 ± 1	0.1	-0.2, 0.4	0.481	0.1	-0.2, 0.2	0.68
Fruit consumption (servings/day)	2 ± 1	2 ± 1	-0.1	-0.4, 0.3	0.686	-0.1	-0.3, 0.2	0.64
Volume of water intake (mL/day)	950 ± 512	966 ± 448	-16	-122, 89	0.756	-30	-138, 79	0.60
Red meat consumption (times/day)	1 ± 1	1 ± 1	0.0	-0.1, 0.2	0.691	0.0	-0.1, 0.1	0.78
**Other eating patterns**								
Breakfast consumption (times/day)	1 ± 0.3	1 ± 0.3	0.04	-0.2, 0.3	0.664	0.06	-0.01, 0.1	0.12
Eating in front of the television (times/day)	0.3 ± 0.4	0.4 ± 0.5	-0.01	-0.2, 0.1	0.700	-0.01	-0.1, 0.1	0.78
**Anthropometry and blood pressure outcomes**								
Weight z-score	0.7 ± 1.1	0.4 ± 1.0	0.3	0.0, 0.5	0.022	0.3	0.0, 0.5	0.04
Height z-score	-0.1 ± 1.0	-0.2 ± 1.0	0.1	-0.1, 0.4	0.248	0.1	-0.1, 0.4	0.36
Arm circumference z-score	1.5 ± 1.1	1.3 ± 0.9	0.2	0.00, 0.5	0.070	0.2	0.1, 0.4	0.17
BMI z score	1.1 ± 1.0	0.9 ± 0.8	0.2	0.00, 0.4	0.019	0.3	0.1, 0.5	0.02
Systolic blood pressure (mmHg)	103 ± 20	103 ± 16	0.6	-3.8, 4.9	0.806	0.4	-4.3, 5.0	0.88
Diastolic blood pressure (mmHg)	67 ± 19	64 ± 14	3	-1, 7	0.183	3	-1, 7	0.17

Abbreviations; BMI, body mass index; CI, confidence interval; MD, mean difference

© 2021 Smithers LG et al. *JAMA Network Open.*

eTable 4. Comparison of Consumers Versus Non-Consumers of Food Categories According to Immediate Intervention (II) Versus Delayed Intervention (DI) Groups at Three Years of Age (Imputed Outcomes)

	II n = 219		DI n = 222		Unadjusted			Adjusted		
	Consumer	Non-consumer	Consumer	Non-consumer	RD	95% CI	P	RD	95% CI	P
Discretionary beverages	92%	8%	96%	4%	-0.04	-0.04, 0.03	<0.001	-0.04	-0.1, 0.01	0.15
Discretionary foods	100%	0%	100%	0%	0[a]	-	-	-	-	-
Diet beverages	19%	81%	20%	80%	-0.01	-0.02, 0.01	0.223	0.00	-0.1, 0.1	0.84
Vegetables	96%	4%	96%	4%	0.00	0.00, 0.01	0.050	0.00	-0.04, 0.04	0.87
Fruit	98%	2%	95%	5%	0.03	0.03, 0.03	<0.001	0.02	-0.02, 0.06	0.45
Milk	89%	11%	89%	11%	0.00	-0.01, 0.01	0.810	0.00	-0.07, 0.06	0.82
Meat	98%	2%	95%	5%	0.02	0.02, 0.03	<0.001	0.02	-0.02, 0.06	0.41

Abbreviations; BMI, body mass index; CI, confidence interval; RD, risk difference

[a] Not possible to compute

© 2021 Smithers LG et al. *JAMA Network Open.*
