# Supplementary material for: Dietary Intake and Anthropometric Measurement at Age 36 Months Among Aboriginal and/or Torres Strait Islander Children in Australia: A Secondary Analysis of the Baby Teeth Talk Randomized Clinical Trial
Source: JAMA Netw Open. 2021 Jul 8;4(7):e2114348. doi: 10.1001/jamanetworkopen.2021.14348 (PMC8267605; doi:10.1001/jamanetworkopen.2021.14348)
Supplement: Supplement 3. — Data Sharing Statement [file jamanetwopen-e2114348-s003.pdf]

Data Sharing Statement

Smithers. Dietary Intake and Anthropometric Measurement at Age 36 Months Among Aboriginal and/or Torres Strait Islander Children in Australia. *JAMA Netw Open*. Published July 08, 2021. doi:10.1001/jamanetworkopen.2021.14348

Data
Data available: No

Additional Information
Explanation for why data not available: Ethical approval for this study does not include publicly posting the study data set. However, de-identified data may be provided to bona fide researchers for a specific research question by contacting the authors.
